# Milk Depots for Infants

**Published:** 1906-01-06

**Authors:** 


### MILK DEPOTS FOR INFANTS.

THE provision of depots at which milk of suitable quality for infants can be purchased, or at which, under proper conditions, it may even be given away, is becoming increasingly prevalent in London; and it may reasonably be hoped that such depots will before long exercise an appreciable effect in the diminution of infantile mortality, and in the prevention of infantile disease. The latest additions to the number have been those of Finsbury and of Lambeth, both of which have been made the subjects of descriptive pamphlets by the respective Medical Officers of Health of the two boroughs. We gather from these pamphlets that the Finsbury depot is primarily a charitable institution, established by the Finsbury Social Workers' Association and by funds privately subscribed; while that of Lambeth has been established by the Borough Council, is supported (so far as it is not self-supporting) by the rates, and furnishes supplies which are available, not only to the poor, but also to every ratepayer who may desire to take advantage of them. In both the object has been to provide for the proper sustenance of infants whose mothers are unable to suckle them; and in both the food supplies are intended to be administered under such supervision as may lead to improved home management; but it is manifest that restrictions tending in this direction may not always be easy of enforcement at Lambeth, where, in the words of the descriptive pamphlet, although the primary object of the Borough Council has been to establish and maintain within the borough a depot for the distribution and sale, at a nominal charge, of specially and suitably prepared (or modified) wholesome pure cow's milk "for the use of infants whose mothers are unable to suckle them wholly or in part," and although instructions have been given that the depot milk shall not be "improperly used, *e.g.* by mothers who are capable of properly and efficiently suckling their offspring, but who simply, for some reason or other, refuse to do so," it is nevertheless found that the depot cannot be entirely so restricted "as *all* Lambeth ratepayers and inhabitants are entitled, if they so wish, to share the benefits to health of such a municipal institution, which, it may be noted, becomes a necessity for the poorer and destitute classes, in that the trade has never attempted, nor offered, to supply specially-prepared (or modified) milk for infants (varying in quantity and quality with their ages) at a price to bring it practically within the reach of such."

In both the new depots, according to the full descriptions given of them, the principles of action are the same. Very strict precautions are enforced with regard to the health and cleanliness both of the cows by which the milk is yielded and of all the members of the staffs in attendance upon them. Immediately after milking, the milk is cooled, strained, and the cream is separated, so as to allow of three different "modifications" being prepared, suitable for infants of different ages. In these modifications water or whey, cream, milk (or cane) sugar, and common salt are added to the pure cow's milk in certain proportions; and the resultant mixture is such as to bear, for the respective ages of the children for whom it is intended, the best attainable imitation of human milk at successive periods after birth. It is then carefully sterilised, and sent out in properly closed bottles, each one of which contains the quantity necessary for a single meal, and which are of a size, and are supplied to each applicant in a daily number, corresponding with the age of the infant to be fed. Endeavours are being made, as far as possible, to keep the infants under the observation of lady visitors, and to have their weights taken and recorded from time to time.

